# Prevalence and associated factors of postoperative nausea and vomiting among adult patients undergoing elective surgery

**DOI:** 10.1097/MS9.0000000000001678

**Published:** 2024-01-05

**Authors:** Sara Timerga, Aynalem Befkadu

**Affiliations:** Department of Anesthesia, College of Medicine and Health Sciences, Wollo University, Dessie, Ethiopia

**Keywords:** Anaesthesia, ethiopia, postoperative nausea and vomiting

## Abstract

**Background:**

Postoperative nausea and vomiting (PONV) is a surgical complication defined as any nausea, and vomiting with in the first 24–48 h after surgery in inpatients. Nausea is the unpleasant desire and urge to vomit, while vomiting is a forcing of gastric contents through the mouth. Nausea and vomiting is the most common complication associated anaesthesia and surgery in the postoperative period. It is considered one of the most common causes of morbidity, and it has significant effects on patient satisfaction.

**Objective:**

The study aimed to assess the incidence and associated factors of postoperative nausea and vomiting.

**Methods:**

A cross-sectional study was conducted from 1 February to 30 April 2022. All adult, elective patients who underwent elective surgery under anaesthesia during the study period were included. A total of 677 patients underwent elective surgery at the time of the study, of which 634 patients were included in the study. Data collection method included chart review and patient interview.

**Result:**

The overall prevalence of postoperative nausea and vomiting among post-surgical patients was 35.4%. Factors that had statistically significant relationship with PONV were history of motion sickness [adjusted odds ratio (AOR) 4.04, 95% CI 1.486–10.988], smoking history (AOR 0.37, 95% CI 0.128–1.042) and intraoperative opioid use (AOR 3.59, 95% CI 1.345–9.618).

**Conclusion:**

The prevalence of this study is higher than studies conducted in the recent years. This result showed that the appropriate practice of PONV prophylactic regimens and anaesthesia management are required to decrease the risk of PONV.

## Background

### Introduction

Highlights

- Postoperative nausea and vomiting (PONV) is a surgical complication defined as any nausea, and vomiting with in the first 24–48 h after surgery in inpatients.
- The prevalence of PONV in DCSH, Ethiopia was observed to be 35.4%.
- Factors that were assessed to be associated with PONV were history of motion sickness, smoking and intraoperative opioid use.

Postoperative nausea and vomiting (PONV) is a surgical complication defined as any nausea, and vomiting with in the first 24–48 h after surgery in inpatients^1^. Nausea is the unpleasant desire and urge to vomit, while vomiting is a forcing of gastric contents through the mouth^2^. Nausea and vomiting is the most common complication associated anaesthesia and surgery in the postoperative period. It is considered one of the most common causes of morbidity, and it has significant effects on patient satisfaction. In spite of recent advancement of perioperative anaesthesia management, the incidence of PONV is reported to be high^1^. This side effect of anaesthetic and surgical has been implicated for the increase patient dissatisfaction and it is believed to be just as stressful for patients as postoperative pain^3^.

The factors associated with postoperative nausea and vomiting vary as; patient related, anaesthesia related and surgery related factors. Patient related factors are factors that steam from the characteristics of the patient; this includes gender, age, smoking status, history of motion sickness, BMI, and history of PONV. The anaesthetics used intraoperatively and postoperatively: volatile anaesthetics, nitrous oxide, large-dose neostigmine, opioids are factors related to anaesthesia, while the type of surgery specialty; obstetric, gynaecologic, ophthalmological, ontological, thyroid surgery, laparoscopic abdominal surgery and duration of the surgery are some of surgery related risk factors^4–6^.

The incidence of PONV for over multiple individual studies conducted around the world has been estimated to be 25–30% and up to 80% in high risk groups^1,7–10^. The incidence of PONV in Ethiopia is reported to range from 20–36%^11–14^.

Postoperative nausea and vomiting has multiple impacts on patients. The leading impact being patient dissatisfaction, but more importantly it leads to dehydration followed by electrolyte imbalance, aspiration of gastric contents, oesophageal trauma, bleeding, and delayed discharge from hospital^15–17^.

In this study, we aimed to assess the incidence and associated factors of postoperative nausea and vomiting, one of the main contributing factor to patient dissatisfaction in the postoperative period and delayed recovery seen in our country. The study was done from February to April 2022 in patients undergoing elective surgery.

## Methods and material

### Study design and period

A cross-sectional study was conducted from 1 February to 30 April 2022.

### Study area

The study was conducted in, north-eastern Ethiopia, Dessie. Dessie city administration is found 401 km away from Addis Ababa, the capital city of Ethiopia. The hospital serves a population of 5 million and has 724 healthcare workers.

The hospital is currently giving multiple services such as medical, surgical, obstetrics and gynaecology, chronic follow-up, paediatric, trauma centre, cough centre and follow-up services, etc. The hospital performs all the major surgical and medical activities. It has nine operation tables, including obstetric surgery. There are six elective surgery tables and three emergency surgery tables.

### Source population

All adult patients who underwent elective surgery under anaesthesia at the hospital.

### Study population

All adult, elective patients who underwent elective surgery under anaesthesia at the hospital during the study period.

### Inclusion criteria

All adult patients, who underwent elective surgery under anaesthesia in the study period, were included.

### Exclusion criteria

patients who underwent emergency surgery, patients who did not recovery from anaesthesia by the time of data collection, patients who underwent reoperation, patients with medical illness predisposing them to nausea and vomiting and patients discharged within 24 h after surgery.

### Dependent variables

Postoperative nausea and vomiting

### Independent variables

Sociodemographic variables (age, sex,), smoking history, previous history of PONV, history of motion sickness, American Society of Anesthesiologists’ (ASA) status, premedication, perioperative use of opioids, type of anaesthesia and anaesthetic drugs used, analgesia and duration of surgery.

### Sample size and sampling procedure

All consecutive patients who underwent elective surgery under anaesthesia during the study period were included.

### Method of data collection

A pilot test was done on 35 patients and changes were made before the data collection.

Data were collected using questionnaires prepared in English and then translated into Amharic. The compatibility of the Amharic version of the questionnaire with the English version was assessed by a group of people who converted it back to English and compared the converted versions. Two recovery nurses were trained how to collect the data for a day by the investigators.

The Amharic questionnaire was used by data collectors to interview patients 24 h after the surgery. From the record sheet and patient chart the type of anaesthesia and anaesthetic drugs, duration of surgery and anaesthesia, ASA classification, perioperative opioid used and premedication were collected.

### Data quality control

A pilot test was done; local language was used; data were cleaned and checked, and double data entry method was employed.

### Data management and analysis

The data were coded, entered and analyzed using SPSS16. The odds ratios and 95% CI, binary and multiple logistic regressions were used to assess the association between dependent and independent variables. Each variable was first analyzed using binary logistic regression to identify the variables that could fit with regression table model (*P* value<0.2), and those variables with *P* value less than 0.2 were entered and further analyzed using multiple logistic regression. Tables used to show the frequency of variables.

### Data analysis

The data were entered into the SPSS version 23 computer program for analysis. Descriptive statistics were summarized; tables and figures were used to display the results. The association between independent variable and the outcome variable was determined by binomial logistic regression and multiple logistic regression analysis; a *P* value of less than 0.05 was considered statistically significant.

The study has been reported in line with the STROCSS criteria^18^

## Results

### Demographic characteristics of study participants

A total of 677 patients underwent elective surgery at the time of the study Table 1. Out of these 677 patients 34 patients were excluded. 14 were excluded due to loss to follow-up, 9 of them were discharged before 24 h, 11 were re-operated on the study period and 3e had a history of motion sickness.

**Table 1:** Demographic status of operated patient.

Variables	Frequency (percentage), *n* (%)
Sex	
Female	309 (48.7)
Male	325 (51.3)
ASA	
ASA I	225 (35.5)
ASA II	405 (63.9)
ASA III	4 (0.6)
Age	
19–38	313 (49.4)
39–57	259 (40.8)
58–77	62 (9.8)
BMI	
Normal	503 (79.2)
Underweight	42 (6.6)
Overweight	89 (14.2)

ASA, American Society of Anesthesiologists.

### Perioperative data

Two hundred ninety six (46.7%) of the patients underwent surgery under general anaesthesia of which 15 (5.1%) patients were induced with thiopental, 84 (28.8%) with Propofol, 148 (50.7%) with ketamine and the rest 43 (15.4%) with ketofol. All patients had prophylactic antibiotics. Overall, 481 (75.8%) of the respondents got intraoperative opioid analgesia. Among which morphine covers the major part, ninety (65.5%). The different surgeries done include; orthopaedics 171 (27%), obstetrics 127 (20%), urology 26 (4%), neurosurgery 30 (4.7%), maxillofacial and ear nose and throat surgery 30 (4.7%), abdominal 214 (33.7%) and gynaecological 45 (7.1%). Of these operations, 590 (93.1%) took more than 60 minutes whereas 44 (6.9%) were below 60 minutes Table 2.

**Table 2:** Factors which affect the prevalence of postoperative nausea and vomiting.

	PONV incidence		
Variables	Yes	No	Percentage (%)
Type of surgery			
Abdominal surgery	127	87	59.3
Orthopaedics	32	139	18.7
Neurologic	12	18	40
Caesarian Section	10	117	7.9
Maxillofacial and ENT	18	12	6
Gynaecology	22	23	48.9
Urology	6	20	23.1
Type of anaesthesia			
GA with ETT	84	212	28.4
SA	143	204	41.2
History of smoking			
Yes	20	121	14.1
No	207	295	41.2
Motion sickness			
Yes	185	39	29.2
No	38	371	5.9
Induction agent			
Propofol	44	40	6.9
Ketamine	29	119	4.6
Thiopental	0	15	0
Ketofol	10	35	1.6
Others	141	201	22.2
Intraoperative opioid use			
Yes	205	276	32.3
No	20	133	3.2
Duration of anaesthesia			
>60 min	207	383	32.6
<60 min	18	26	2.8
Sex			
Female	142	167	22.4
Male	83	242	13
ASA			
ASA I	76	149	12
ASA II	149	256	23.5
ASA III	0	4	0
BMI			
Normal	187	316	29.5
Underweight	0	42	0
Overweight	38	51	5.9

ASA, American Society of Anesthesiologists; ENT, ear nose and throat surgery; ETT, endotracheal tube; GA, general anesthesia; PONV, postoperative nausea and vomiting.

### Prevalence of postoperative nausea and vomiting

The overall prevalence of postoperative nausea and vomiting at the Dessie comprehensive hospital among post-surgical patients was 35.4% Table 3. Out of these patients 108 (17%) patients suffered from both nausea and vomiting. Out of 634 patients, 409 (64.5%) had no complications. The overall prevalence of postoperative nausea was 164 (25.4%), and nausea without vomiting occurred in 56 (8.8%) patients. The overall prevalence of postoperative vomiting was 169 (26.7%), and vomiting without nausea occurred in 61 patients (27.1%) Table 4.

**Table 3:** Prevalence of PONV.

	Frequency (percent), *n* (%)
Postoperative nausea and vomiting	
Yes	225 (35.4)
No	409 (64.6)

PONV, postoperative nausea and vomiting.

**Table 4:** Postoperative nausea and vomiting distribution.

	Vomiting		
Nausea	No	Yes	Total
No	409	61	470
Yes	56	108	164
Total	465	169	634

### Factors associated with postoperative nausea and vomiting

The bivariate analysis for this study showed that previous history of PONV, history of motion sickness, non-smokers, type of surgery, duration of surgery, intraoperative opioid use and intraoperative fluid given amount were found to be statistically significant relationship with postoperative nausea and vomiting with a *P* value less than 0.2 Table 5. These variables were taken for multivariate analysis which is crucial to see the strength of association. As shown on Table 5, variables which are strongly associated with PONV are: previous history of smoking, motion sickness and intraoperative opioid use.

**Table 5:** Factors associated with postoperative nausea (PONV): results of bivariate logistic regression analysis.

Variables	PONV incidence	Percentage (%)	AOR (95% CI)	*P*
History of smoking				
Yes	22	9.8	10.62 (1.08–104)	0.042
No	203	90.2		
Motion sickness				
Yes	187	83.1	0.002 (0.000–0.018)	0.000
No	38	16.9		
PONV history				
Yes	159	70.6	0.226 (0.056–0.909)	
No	65	29.4		
Intraoperative opioid use				
Yes	205	91.1	0.053 (0.007–0.397)	0.04
No	20	8.9		
Duration of anaesthesia				
>60 min	207	92	0.22 (0.025–1.95)	0.174
<60 min	18	8		
Type of anaesthesia				
GA with ETT	83	36.8	0.038 (0.001–1.86)	0.099
SA	142	63.2		
Intraoperative fluid given				
<500 ml	4	1.8		0.206
500 –1000ml	10	4.4		0.999
1000 –2000ml	153	68		0.358
>2000 ml	58	25.8	0.053 (0.007–0.397)	0.040

AOR, adjusted odds ratio; ETT, endotracheal tube; GA, general anesthesia; PONV, postoperative nausea and vomiting.

According to the multivariate analysis, patients with history of smoking have the odds of developing PONV 63% lower than those patients who have no history of smoking with adjusted odds ratio of 0.37, CI (0.128–1.042) Table 6. Patients with history motion sickness are 4.04 times more likely to develop PONV than those without any known history of motion sickness with CI (1.486–10.988). Patients with intraoperative opioid use for analgesia had 3.59 times higher chance of developing PONV than those patients who didn’t take any opioid in the intraoperative period with CI (1.345–9.618).

**Table 6:** Factors associated with postoperative nausea (PONV): results of multivariate logistic regression analysis.

Variables	PONV incidence	Percentage (%)	COR (95% CI)	*P*
History of smoking				
Yes	22	9.8	0.37 (0.128–1.042)	0.05
No	203	90.2		
Motion sickness				
Yes	187	83.1	4.04 (1.486–10.988)	0.006
No	38	16.9		
Intraoperative opioid use				
Yes	205	91.1	3.59 (1.345–9.618)	0.01
No	20	8.9		

COR, crude odds ratio; PONV, postoperative nausea and vomiting.

## Discussion

This study showed that the prevalence of postoperative nausea and vomiting (PONV) was 35.4%. This finding is comparable with previous studies conducted in the same study setting^11–14^. The high incidence of PONV is due to the practice of not using nausea and vomiting prophylaxis in the preoperative period combined with the use of high dose opioids as solo analgesics.

The result of our study showed higher incidence compared to other studies conducted in the recent years^12,19^, that can be explained by the prophylactic medication use and anaesthesia management of practice in the hospital. There was no use of prophylactic premedication trend and the analgesics provided for the patients were opioids alone with higher dose than usual to accommodate the analgesic needs of individual patients.

Our research showed that, previous history of motion sickness and smoking along with intraoperative opioid use had statistically significant association with PONV. Patients with history of motion sickness had four times higher risk of developing PONV, while patients with history of smoking showed a protective against PONV with AOR of 0.37. This finding corresponds with studies conducted in Ethiopia 2019^12^, in south Korea 2017^20^ and in USA 2012^21^. The other factor we found was patients who had taken opioid for intraoperative analgesia had 3.5 times higher risk of developing PONV compared to patients who underwent surgery without opioid analgesia. This finding is supported by studies conducted in USA 2016^8^, in china 2023^22^, in Republic of Macedonia 2022^23^.

Other factors that have been known to be a risk factor for PONV like female sex^9,10,24^, duration of anaesthesia and surgery^24^, type of surgery^25^, previous history of PONV^10,24^ and amount of intraoperative fluid administered^13^, we found no significant association with PONV. The reason could be the fact that we have included all type of surgery and anaesthesia in the study population which could result in a diminished effect of these factors on PONV.

### Limitations of the study

The limitation of our study, the study population is not specific to surgery type and anaesthesia type. The study period was for only 24 h.

## Conclusion

The prevalence of our study is higher than studies conducted in the recent years after the identification of the risk factors associated with PONV. This can be explained by the clinical practice of Dessie comprehensive hospital at the moment. There are no prophylactics measure taken to prevent the complication and the APFEL risk factors are not considered in the anaesthesia management of patients.

### Recommendations

The recommendation we provide based on our result is the appropriate practice of PONV prophylactic regimens and anaesthesia management based on the ERAS protocol^26^. The risk assessment based on the listed risk factors should be conducted for each patient.

## Ethics approval and consent to participate

An approval letter from Departmental Research and Ethics Review Committee and patients’ written informed consents was obtained. A copy of the written consent is available for review by the Editor-in-Chief of this journal on request.

## Consent for publication

Not applicable.

## Source of funding

Wollo University is the source of funding for this study.

## Author contribution

S.T. wrote the proposal, manuscript, and cover letter, drafted the questionnaire, and assessed the data quality was involved in the analysis and data presentation. A.B. wrote the manuscript and was involved in data analysis and presentation Both authors reviewed the manuscript and cover letter.

## Conflicts of interest disclosure

There are no potential conflicts of interest with respect to the research, authorship, and/or publication of this article.

## Research registration unique identifying number (UIN)

1. Registry used- ClinicalTrials.gov

2. Unique Identifying number or registration ID- NCT05970055

3. Hyperlink to your specific registration- https://ichgcp.net/clinical-trials-registry/NCT05970055

## Guarantor

Sara Timerga.

## Data availability statement

All data generated and analysed during this study are available to be provided if asked for them.

## Provenance and peer review

This paper is not invited.
